# The “Balgrist Score” for evaluation of Charcot foot: a predictive value for duration of off-loading treatment

**DOI:** 10.1007/s00256-020-03541-6

**Published:** 2020-07-23

**Authors:** Martin C. Berli, Kai Higashigaito, Tobias Götschi, Christian W. A. Pfirrmann, Reto Sutter, Andrea B. Rosskopf

**Affiliations:** 1 Orthopedic Surgery, Balgrist University Hospital, Forchstrasse 340, CH-8008 Zurich, Switzerland; 2 Faculty of Medicine, University of Zurich, Zurich, Switzerland; 3 Radiology, Balgrist University Hospital, Forchstrasse 340, CH-8008 Zurich, Switzerland; 4 Unit for Clinical and Applied Research (UCAR), Balgrist University Hospital, Forchstrasse 340, CH-8008 Zurich, Switzerland; 5 Institute for Biomechanics, Swiss Federal Institute of Technology, Zurich, Switzerland

**Keywords:** Charcot foot, Conservative treatment, Neuropathic arthropathy, Diabetic foot, MR imaging

## Abstract

**Objective:**

To develop a new magnetic resonance imaging(MRI) scoring system for evaluation of active Charcot foot and to correlate the score with a duration of off-loading treatment ≥ 90 days.

**Methods:**

An outpatient clinic database was searched retrospectively for MRIs of patients with active Charcot foot who completed off-loading treatment. Images were assessed by two radiologists (readers 1 and 2) and an orthopedic surgeon (reader 3). Sanders/Frykberg regions I–V were evaluated for soft tissue edema, bone marrow edema, erosions, subchondral cysts, joint destruction, fractures, and overall regional manifestation using a score according to degree of severity (0–3 points). Intraclass correlations (ICC) for interreader agreement and receiver operating characteristic analysis between MR findings and duration of off-loading-treatment were calculated.

**Results:**

Sixty-five feet in 56 patients (34 men) with a mean age of 62.4 years (range: 44.5–85.5) were included. Region III (reader 1/reader 2: 93.6/90.8%) and region II (92.3/90.8%) were most affected. The most common findings in all regions were soft tissue edema and bone marrow edema. Mean time between MRI and cessation of off-loading-treatment was 150 days (range: 21–405). The Balgrist Score was defined in regions II and III using soft tissue edema, bone marrow edema, joint destruction, and fracture. Interreader agreement for Balgrist Score was excellent: readers 1/2: ICC 0.968 (95% CI: 0.948, 0.980); readers 1/2/3: ICC 0.856 (0.742, 0.917). A cutoff of ≥ 9.0 points in Balgrist Score (specificity 72%, sensitivity 66%) indicated a duration of off-loading treatment ≥ 90 days.

**Conclusion:**

The Balgrist Score is a new MR scoring system for assessment of active Charcot foot with excellent interreader agreement. The Balgrist Score can help to identify patients with off-loading treatment ≥ 90 days.

**Electronic supplementary material:**

The online version of this article (10.1007/s00256-020-03541-6) contains supplementary material, which is available to authorized users.

## Introduction

The Charcot foot is a potentially devastating complication of patients with peripheral polyneuropathy leading to considerable bone destruction, foot deformity, and risk of pedal ulcer formation [1–4]. The disease affects the bones, joints, and soft tissues of the foot and ankle. Several theories exist regarding the multifactorial etiology of this disease, including repetitive microtrauma and increased blood flow [5, 6]. The prevalence of a Charcot foot in diabetic patients with apparent peripheral neuropathy is up to 35%, regardless of the type of diabetes (i.e., type I or II) [7]. In the early disease stage, an acute (i.e., active) Charcot foot shows major signs of inflammation, including redness, edema, and hyperthermia—overlapped by subsequent stages of bone fragmentation and joint destruction. A late-stage (i.e., chronic) Charcot foot shows signs of consolidation in order to repair the earlier changes. Untreated, the typical end shape of a Charcot foot is the so-called rockerbottom deformity [8]. Patients often perceive their quality of life as poor, since a foot with rockerbottom deformity cannot be equipped with commercial footwear, making ulcer formations and infections very likely. If left untreated, a Charcot foot can become a limb-threatening condition, as the ulcerations can cause cellulitis or osteomyelitis, and may require amputation [6, 9]. Anatomical and imaging-based systems are available for Charcot foot classification, the most common ones being the Brodsky classification [10] (modified by Trepman et al. [11]), the Sanders/Frykberg classification [12], and the Eichenholtz classification [13] (modified by Shibata et al. [14]). These classifications rely on conventional radiographs for disease evaluation. Although magnetic resonance imaging (MRI) has become an essential tool for early disease diagnosis, monitoring of treatment success, and detection of complications, no established MRI-based classification system is currently available [4, 13–15]. The two major treatment options for active Charcot foot include early surgical treatment with joint fusions (i.e., arthrodesis) or traditional conservative treatment with off-loading therapy of the affected foot [15]. The off-loading treatment should start as early as possible, such that the inflammatory and destructive disease stages can proceed while the foot is protected from major shape changes [16, 17]. Patients are frequently treated with a custom-made, removable, total contact cast until the signs of active Charcot foot are substantially reduced or absent [4]. Off-loading therapy can last up to 18 months [18]. Individual treatment duration is dependent on various signs of disease regression assessed by both clinical examination and MRI including reductions and elimination of edema in bones and soft tissues [4, 19]. Our clinical experience is that patient compliance in the first 3 months (90 days) of off-loading is very good, while longer treatment durations cause major frustrations. No imaging parameter is currently available to predict the approximate duration of off-loading therapy, especially in order to help prepare patients for a treatment longer than 3 months (90 days).

The purpose of this study was to develop a new non-contrast MRI scoring system for the evaluation of active Charcot foot and to assess if this score can be used to predict a duration of off-loading therapy ≥ 90 days.

## Materials and methods

This retrospective, observational, uncontrolled cohort study was approved by the local Ethics Committee Zurich, Switzerland (No. ZH-2016-00271).

PACS and electronic medical records at the outpatient clinic of a large, urban, orthopedic, university-affiliated research hospital were searched for adult patients (i.e., age > 18 years) with a diagnosis of active Charcot foot with conservative off-loading treatment between July 2014 and December 2018.

The diagnosis of “Charcot foot” was made by an interdisciplinary team of orthopedic surgeons, neurologists, and radiologists in all cases. The Charcot foot was defined as “active” by the clinicians when redness, swelling, and hyperthermia were present at the time of MRI examination.

Off-loading had to be completed at least 6 months earlier with no known follow-up complications (i.e., infections, operation, or re-activation).

MR images: The first available MRI examination after diagnosis, whether conducted at our institution or elsewhere, was used. The images had to show the whole foot, with at least two fluid-sensitive sequences (at least one of them with fat-saturation), and at least two T1-weighted sequences. The Balgrist standard protocol can be found in the Supplemental Material (Fig. S1).

Exclusion criteria for the study were poor MR image quality (e.g., artifacts, missing sequences), clinically inactive Charcot arthropathy, other inflammatory diseases (e.g., osteomyelitis, soft tissue infection, infected foot ulcers, complex regional pain syndrome (CRPS), gout, rheumatoid arthritis), documented non-compliance during off-loading treatment, and immunosuppressive medication.

### Image analysis

**Fig. 1:**
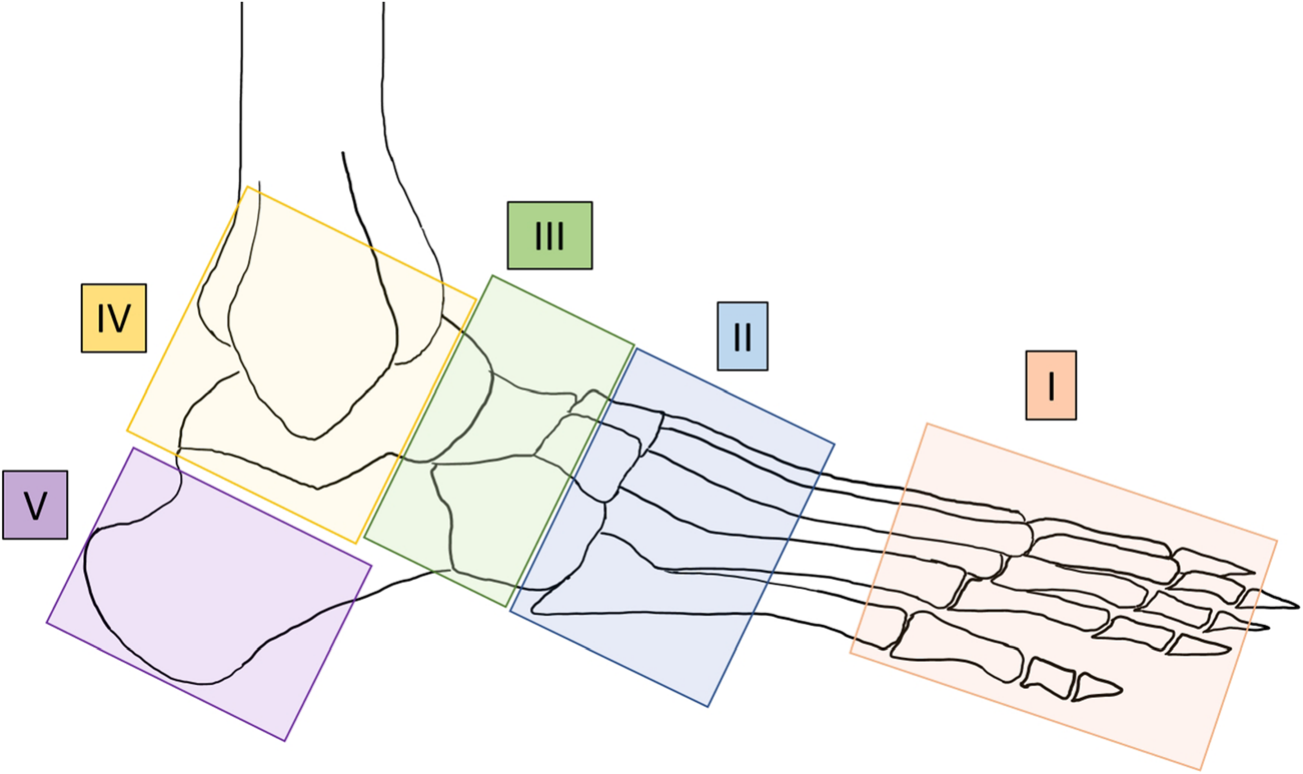
Regions I to V (adapted from Sanders and Frykberg [3, 12])

**Fig. 2:**
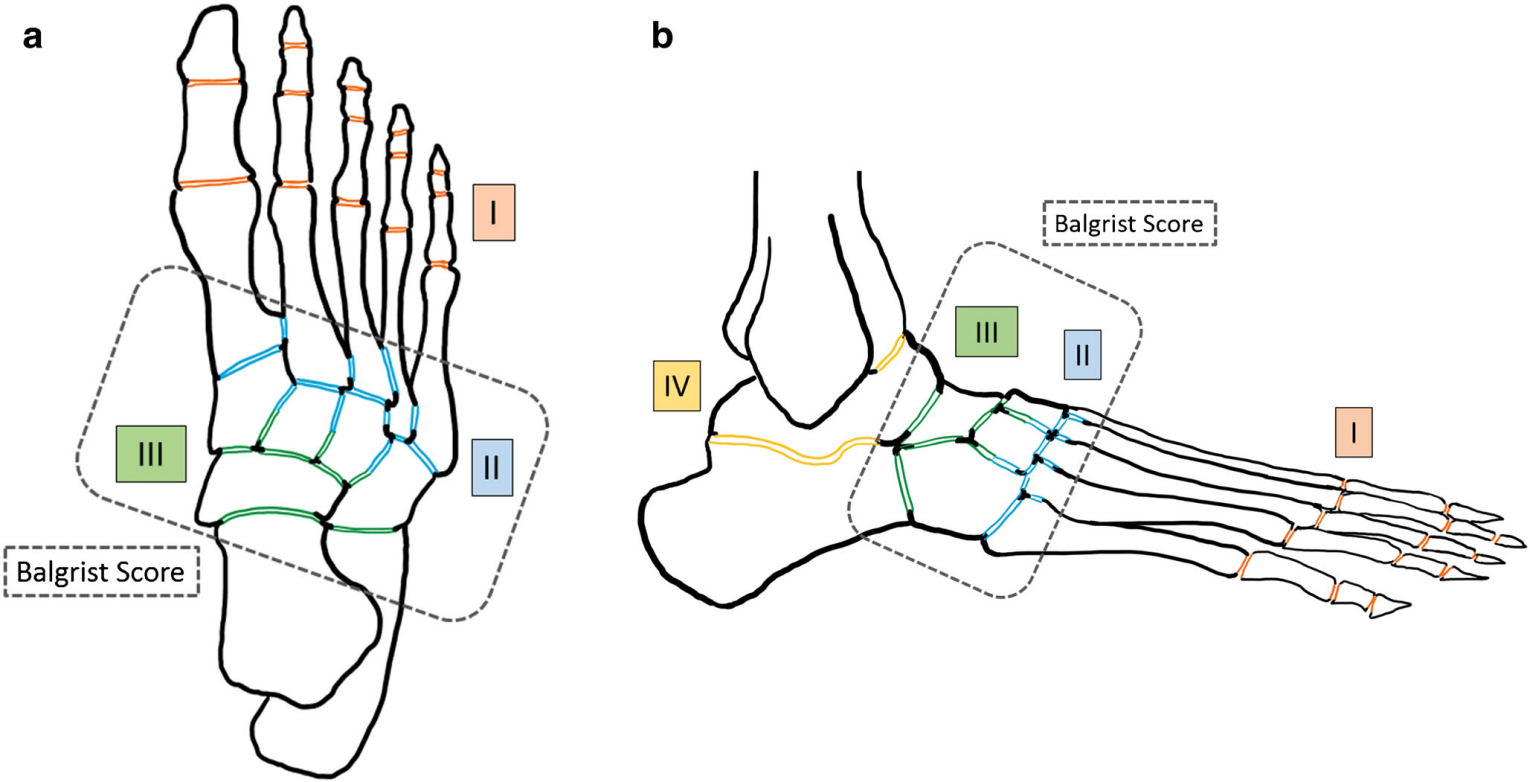
Transverse (**a**) and lateral view (**b**) of foot showing the regional joint surface areas (according to Sanders/Frykberg classification) that were searched for erosions, subchondral cysts, and joint destruction according to the following scheme: 0 = 0%, 1 = 1–33%, 2 = 34–66%, 3 = 67%–100% (of total surface area per region). Areas included in the Balgrist Score are circled with a dotted line

The MR images were assessed by three readers: two fellowship-trained radiologists (reader 1 = ABR and reader 2 = KH) with 9 and 2 years of experience in musculoskeletal radiology, respectively, and an orthopedic surgeon (reader 3 = MCB) with 10 years of experience in Charcot foot therapy. All readers were blinded to the patients’ data and clinical information. All five anatomic regions of the foot according to the Sanders and Frykberg classification (Fig. 1) were evaluated for the presence of soft tissue edema, erosions, bone marrow edema, subchondral cysts, fracture, joint destruction, and overall regional manifestation using fluid-sensitive and T1-weighted sequences. Since region V does not include any joint surface, only soft tissue edema, bone marrow edema, fracture, and overall regional manifestation were assessed in this region. Detailed parameters assessed in the MRI readout are presented in Fig. 2 and Table 3. Detailed classification of soft tissue edema is provided in the Supplemental Material (Fig. S2). Severity of erosions, subchondral cysts, and joint destruction was evaluated per region according to the affected proportion of the involved joint surface area, where 0 = 0%, 1 = 1–33%, 2 = 34–66%, and 3 = 67–100% (Fig. 2). For bone marrow edema, all bones in a region were seen as a whole block and classified in a similar way: no edema, where 1 = 1–33% of bones with edema, 2 = 34–66% of bones with edema, and 3 = 67–100% of bones with edema. Additionally, reader 1 evaluated all cases with bone marrow edema with respect to the signal intensity of the bone marrow edema on the fluid-sensitive (with fat-suppression) and T1-weighted sequences. Low signal intensity was defined as hyperintense on the fluid-sensitive sequence without a signal drop on the corresponding T1-weighted sequence. High signal intensity was defined as hyperintense on the fluid-sensitive sequence and hypointense on the corresponding T1-weighted sequence.

### Statistical analysis

Descriptive statistics were determined using Microsoft Excel 2010. Interclass correlations (ICC) and Fleiss’ kappa were calculated for interreader reliability using Matlab (release 2019b, The MathWorks, Inc., Natick, Massachusetts, USA). The same software was used for calculation of Spearman rank correlation, receiver operator characteristics (ROC), and area under the curve (AUC). ICC values were interpreted as follows: less than 0.40 = poor, between 0.40 and 0.59 = fair, between 0.60 and 0.74 = good, between 0.75 and 1.00 = excellent. Differences of *p* < 0.05 were considered statistically significant.

## Results

### Patient population

Sixty-five Charcot feet in 56 patients (34 men, 22 women) were included. Both feet were included in 5 men and 4 women. Mean age and standard deviation (SD) of patients was 62.4 ± 9.6 years. Mean duration of off-loading treatment was 150 days ± 95 SD (range: 21–405 days). The treatment duration was < 90 days in 18 feet (18 patients) and ≥ 90 days in 47 feet (38 patients). Diabetes was present in 10 (55.6%) patients with off-loading duration < 90 days and in 25 (65.7%) patients with off-loading duration ≥ 90 days. Further patients’ characteristics are summarized in Table 1.

**Table 1:** Patients’ characteristics

	*n*
Feet	65
Patients	56
Age, years; mean (range)	62.4 (44.5–85.5)
Gender f/m	22/34
Diabetes	35
Diabetes type I/II	2/33
Alcoholism	3
Cobalamin deficiency (Vit. B12)	5
Hepatitis B and C	1
HIV	1
Chemotherapy	3
Autoimmune disease	4
Adipositas with BMI ≥ 35	4
Peripheral artery disease	9
Kidney failure or transplantation	11
Gout	6

### Interreader agreement

Interreader agreement for the two radiologists was excellent with ICC values between 0.707 and 979 (Table 2). Interreader agreement between all three readers was excellent with ICC values between 0.814 and 0.916, with the exception of region V (ICC = 0.396, Table 2).

**Table 2:** Interreader agreements

	ICC (95% CI) readers 1 and 2	ICC (95% CI) readers 1, 2, and 3
Region I	0.914 (0.864, 0.947)	0.814 (0.634, 0.899)
Region II	0.979 (0.965, 0.987)	0.916 (0.877, 0.945)
Region III	0.950 (0.919, 0.969)	0.910 (0.870, 0.941)
Region IV	0.966 (0.945, 0.978)	0.901 (0.853, 0.935)
Region V	0.707 (0.553, 0.813)	0.396 (0.245, 0.546)
Balgrist Score	0.968 (0.948, 0.980)	0.856 (0.742, 0.917)

ICC (Interclass correlation), mixed model, based on single measure, absolute agreement. Reader 1 = radiologist (ABR), reader 2 = radiologist (KH), reader 3 = orthopedic surgeon (MCB). More detailed interreader agreements per parameter (item) can be found in the Supplemental Material

*CI* confidence interval

Detailed interreader agreements per item for readers 1 and 2 are provided in the Supplemental Material (Table S1): Fleiss’ kappa values were between 0.467 and 1.000.

### MRI readout

The most common finding in all regions was soft tissue edema, followed by bone marrow edema, bone erosions (regions I–IV), and subchondral cysts (regions I–IV). All readout findings of readers 1 and 2 are listed in Table 3. The detailed results of reader 3 are not shown for the sake of readability.

**Table 3:** Results MR readout readers 1 and 2

*n* = 65	Score	*n* (%) Reader 1/reader 2				
		Region I	Region II	Region III	Region IV	Region V
Soft tissue edema						
None	0	19 (29.2)/15 (23.1)	2 (3.1)/2 (3.1)	2 (3.1)/2 (3.1)	21 (32.3)/23 (35.4)	51 (78.5)/58 (89.2)
Mild	1	29 (44.6)/33 (50.8)	22 (33.8)/21 (32.3)	32 (49.2)/33 (50.8)	37 (56.9)/35 (53.8)	12 (18.5)/5 (7.7)
Moderate	2	9 (13.8)/9 (13.8)	19 (29.2)/22 (33.8)	23 (35.4)/22 (33.8)	6 (9.2)/5 (7.7)	2 (3.1)/2 (3.1)
Severe	3	8 (12.3)/8 (12.3)	22 (33.8)/20 (30.8)	8 (12.3)/8 (12.3)	1 (1.5)/2 (3.1)	0/0
Bone erosion						
None	0	60 (92.3)/62 (95.4)	14 (21.5)/13 (20.0)	24 (36.9)/24 (36.9)	55 (84.6)/53 (81.5)	NA
1–33%	1	5 (7.7)/3 (4.6)	22 (33.8)/20 (30.8)	24 (36.9)/24 (36.9)	8 (12.3)/6 (9.2)	NA
34–66%	2	0/0	10 (15.4)/13 (20.0)	11 (16.9)/11 (16.9)	1 (1.5)/5 (7.7)	NA
67–100%	3	0/0	19 (29.2)/19 (29.2)	6 (9.2)/6 (9.2)	1 (1.5)/1 (1.5)	NA
Bone marrow edema						
None	0	38 (58.5)/48 (73.8)	5 (7.7)/8 (12.3)	3 (4.6)/4 (6.2)	29 (44.6)/32 (49.2)	52 (80.0)/57 (87.7)
1–33%	1	25 (38.5)/15 (23.1)	23 (35.4)/23 (35.4)	24 (36.9)/22 (33.8)	26 (40.0)/21 (32.3)	13 (20.0)/8 (12.3)
34–66%	2	2 (3.1)/2 (3.1)	13 (20.2)/11 (16.9)	25 (38.5)/27 (41.5)	7 (10.8)/9 (13.8)	0/0
67–100%	3	0/0	24 (36.9)/23 (35.4)	13 (20.2)/12 (18.5)	3 (4.6)/3 (4.6)	0/0
Subchondral cysts						
None	0	63 (96.9)/62 (95.4)	37 (56.9)/35 (53.8)	33 (50.8)/34 (52.3)	53 (81.5)/54 (83.1)	NA
1–33%	1	2 (3.1)/3 (4.6)	26 (40.0)/28 (43.1)	30 (46.2)/28 (43.1)	11 (16.9)/10 (15.4)	NA
34–66%	2	0/0	2 (3.1)/2 (3.1)	2 (3.1)/3 (4.6)	1 (1.5)/1 (1.5)	NA
67–100%	3	0/0	0/0	0/0	0/0	NA
Joint destruction						
None	0	61 (93.8)/63 (96.9)	26 (40.0)/27 (41.5)	31 (47.7)/26 (40.0)	55 (84.6)/54 (83.1)	NA
1–33%	1	4 (6.2)/2 (3.1)	10 (15.4)/8 (12.3)	18 (27.7)/23 (35.4)	7 (10.8/7 (10.8)	NA
34–66%	2	0/0	14 (21.5)/14 (21.5)	9 (13.8)/8 (12.3)	2 (3.1)/3 (4.6)	NA
67–100%	3	0/0	15 (23.1)/16 (24.6)	7 (10.8)/8 (12.3)	1 (1.5)/1 (1.5)	NA
Fracture*						
None	0	61 (93.8)/61 (93.8)	59 (90.8)/60 (92.3)	63 (96.9)/62 (95.4)	64 (98.5)/64 (98.5)	65 (100)/65 (100)
Yes	3	4 (6.2)/4 (6.2)	6 (9.2)/5 (7.7)	2 (3.1)/3 (4.6)	1 (1.5)/1 (1.5)	0/0
Regional manifestation						
None	0	52 (80.0)/58 (89.2)	5 (7.7)/6 (9.2)	4 (6.2)/6 (9.2)	45 (69.2)/50 (76.9)	59 (90.8)/63 (96.9)
Mild	1	10 (15.4)/4 (6.2)	21 (32.3)/21 (32.3)	30 (46.2)/31 (47.7)	12 (18.5)/8 (12.3)	6 (9.2)/2 (3.1)
Moderate	2	3 (4.6)/3 (4.6)	14 (21.5)/15 (23.1)	22 (33.8)/18 (27.7)	5 (7.7)/5 (7.7)	0/0
Severe	3	0/0	25 (38.5)/23 (35.4)	9 (13.8)/10 (15.4)	3 (4.6)/2 (3.1)	0/0

* defined as clearly visible fracture line outside the joint surface area

Soft tissue edema was most common in region II (reader 1/reader 2 = 96.6/96.6%) and region III (96.6/96.6%) and least common in region V (21.5/10.8%). Severe soft tissue edema was more common in region II (33.8/30.8%) than III (12.3/12.3%).

Bone erosions were most commonly seen in region II (78.5/80.0%) and region III (63.1/63.1%) and were least common in region I (7.7/4.6%) and region V. Bone erosions covering more than 66% of the regional joint surface area were most frequently seen in region II (29.2/29.2%) and region III (9.2/9.2%).

Bone marrow edema was most common in regions II (reader 1/reader 2 = 95.4/93.8%) and III (92.3/87.7%) and least common in region V (20/12.8%). Severe bone marrow edema covered more than 66% of regional bone surface area in region II (36.9/35.4%), followed by region III (20.2/18.5%) and region IV (4.6/4.6%).

Signal intensity of bone marrow edema assessed by reader 1 was as follows:

In region I, of 27 feet with bone marrow edema, 23 feet (= 85.2%) showed edema with low signal intensity, and 4 feet (14.8%) showed edema with high signal intensity. In region II, 31 of 60 feet (51.7%) showed low signal intensity, and 29 of 60 feet (48.3%) showed high signal intensity. In regions III/IV/V of 62/36/13 feet with bone marrow edema, low signal intensity was seen in 30/28/11 feet (= 48.4/77.7/84.6%), and high signal intensity was seen in 32/8/2 feet (51.6/22.3/15.4%).

Subchondral cysts were most common in region III (reader 1/reader 2 = 49.2/47.7%), followed by region II (43.1/46.2%) (Table 3). Subchondral cysts were largely observed in less than 34% of the regional joint surface area (i.e., regions II and III) and never affected more than 66% of the joint surface area.

Fractures were rarely observed: in 4 cases (reader 1 and 2) in region I, in 6 (reader 1) or 5 (reader 2) cases in region II, in 2 (reader 1) or 3 (reader 2) cases in region III, and in 1 case (reader 1 and 2) in region IV. No fracture occurred in region V.

Joint destruction occurred most often in region II (reader 1/reader 2: 60.0/58.5%), region III (52.3/60.0), and region IV (15.4/16.9%). The most cases with severe joint destruction (i.e., more than 66% of joint surface area) were seen in region II, in 23.1/24.6% of feet.

The regions of the foot most commonly affected by Charcot disease (i.e., regional manifestation) were region III (93.6/90.8%) and region II (92.3/90.8%), followed by region I (20/10.8%). The least commonly affected area was region V. Severe regional manifestation was highest in region II affecting 38.5/35.4% of feet and lowest in region V (only cases with mild manifestation).

### Correlation between MRI score and treatment duration

No significant correlation was found between the intensity of bone marrow edema in the five regions (assessed by reader 1) and duration of off-loading treatment: *r*-values of Spearman’s rank correlation were between − 0.05 and 0.06, *p* values between 0.638 and 0.991.

The Spearman rank correlation (*r*-value) of each MR parameter with duration of off-loading therapy was between − 0.13 and 0.37 (*p* 0.002–0.980) for reader 1 and between − 0.18 and 0.32 (*p* 0.009–0.890) for reader 2, showing only weak correlations.

**Fig. 3:**
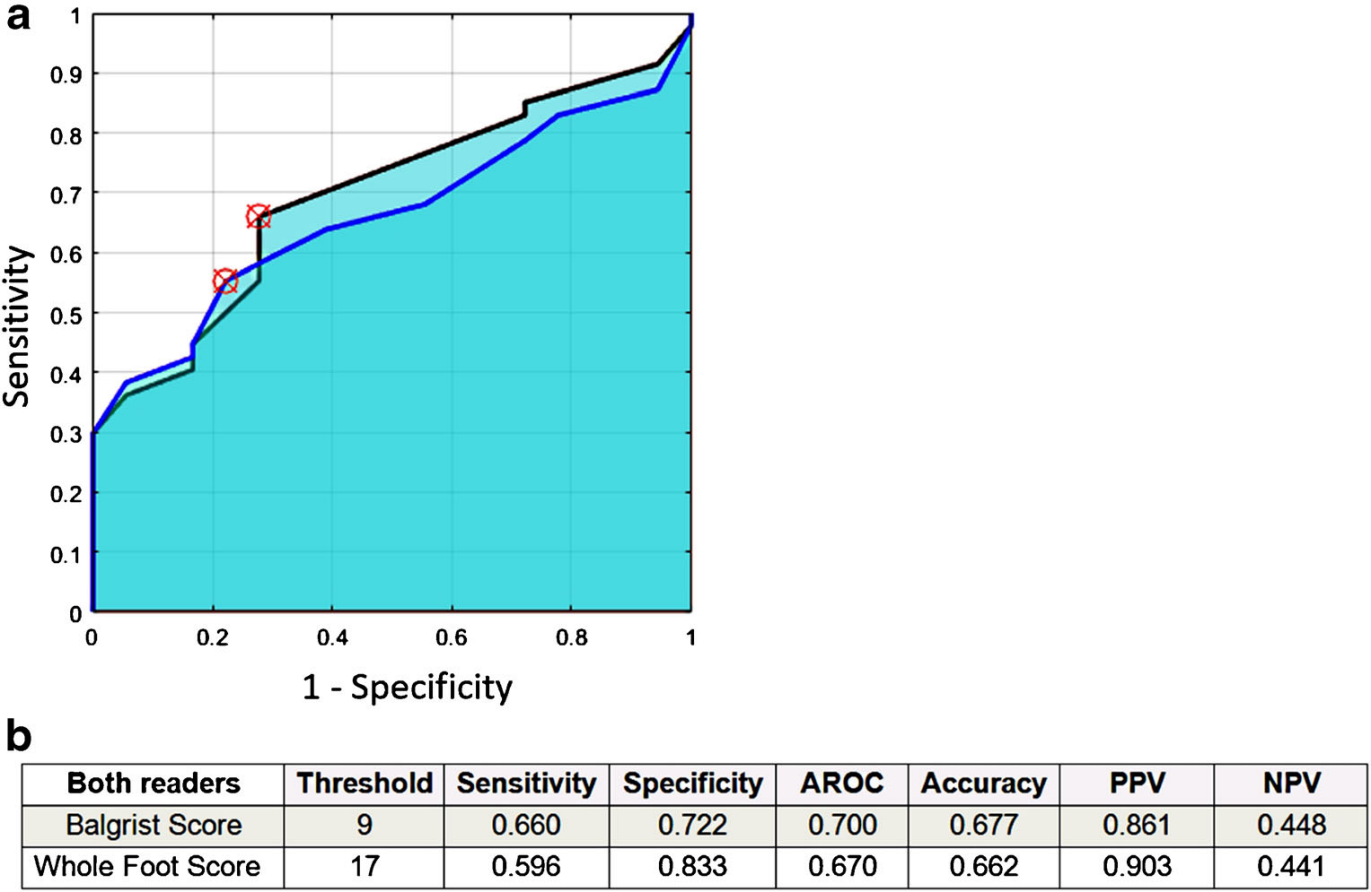
**a** The receiver operator characteristics (ROC) of reader 1 (black line) and reader 2 (blue line) for the Balgrist Score. The optimal threshold for prediction of off-loading treatment ≥ 90 days (red cross) was chosen based on the distance of each point in the ROC curve to the point with 100% sensitivity and 100% specificity. The table **b** shows the values in detail. **b** Results of MRI Scores for both readers. AROC, area under the ROC curve; PPV, positive predictive value; NPV, negative predictive value. Threshold = points

All MR parameters were additionally scored with points according to the scheme in Table 3. The mean total score for the whole foot was 18.1 points (SD ± 7.4, range 6–35) for reader 1 and 17.5 points (SD ± 7.7, range 5–36) for reader 2. Scoring of the whole foot (“Whole Foot Score”) resulted in a sensitivity of 60% and specificity of 83% for the detection of off-loading treatment ≥ 90 days when using a cutoff value of 17 points. Detailed results for this Whole Foot Score are found in Fig. 3b.

### Balgrist Score

Since a detailed scoring of the whole foot is not practicable in daily routine, we developed a simplified score. The “Balgrist Score” (Table 4; Fig. 2) includes changes in only the most frequently affected regions of the foot, i.e., regions II and III, and focuses on the four essential parameters, which demonstrated some significant correlations as single parameters in the Spearman rank correlation: soft tissue edema, bone marrow edema, joint destruction, and fracture.

**Table 4:** Balgrist Score

Balgrist Score				
	Region	II	III	Total (points)
Finding	Scoring of findings (points)			
Soft tissue edema*	0 = none, 1 = mild, 2 = moderate, 3 = severe			
Bone marrow edema	0 = none, 1 = 0–33%, 2 = 33–66%, 3 = 67–100%			
Fracture	0 = none, 3 = present			
Joint destruction	0 = none, 1 = 0–33%, 2 = 33–66%, 3 = 67–100%			
Total (maximum 24 points)				
≥ 90 days off-loading				≥ 9.0

*Example of soft tissue edema classification can be found in the Supplemental Material (Fig. S2)

Using these parameters, the mean Balgrist Score in all feet was 9.6 points (SD ± 3.8, range 3–18) for reader 1 and 9.6 points (SD ± 3.9, range 3–17) for reader 2. A cutoff value of ≥ 9 points for the Balgrist Score was found to predict an off-loading duration of ≥ 90 days (Fig. 3) with a specificity of 72% and a sensitivity of 66%.

## Discussion

The new MRI scoring system (“Balgrist Score”) for patients with active Charcot foot described here showed excellent interreader reliability. A Balgrist Score ≥ 9 points predicted a duration of off-loading treatment ≥ 90 days in patients with an active Charcot foot.

To our knowledge, no other study has investigated a correlation between imaging findings and treatment duration. A recent study by Chantelau et al. [19] demonstrated that conventional MRI is a useful tool for surveillance of active-stage Charcot foot recovery, but no correlation with treatment duration was performed. Rettedal et al. [20] introduced a clinical scoring system (Charcot Reconstruction Preoperative Prognostic Score) as a starting point for treatment decisions, but no imaging parameters were included. Meacock et al. [21] proposed a scoring system for bone marrow edema and fracture assessment for each pedal bone in patients with Charcot foot, wherein a maximum score of 44 points (each for edema and fracture) could be reached. Unfortunately, the authors [21] did not provide a detailed distribution of bone marrow edema for comparison with our study findings. Since a separate scoring of each individual bone is very time consuming, we chose to assess the different regions of the foot (I to V) according to the classification of Sanders and Frykberg. The most commonly affected region in our study was region III, followed by region II, in which the manifestation demonstrated a higher degree of severity. The lowest manifestation of Charcot disease was seen in region V. This distribution pattern with focus on regions II and III has been reported in studies by Chantelau and Poll [22] and by Sella and Barrette [23] and is also mentioned in reviews [3, 4]. We chose a separate evaluation of the parameter “regional manifestation” because this parameter summarizes the severity of clearly Charcot-related changes per region: All other findings that might not be caused by Charcot disease (e.g., bone marrow edema due to stress reaction, osteoarthritis, or vascular diseases) were neglected for evaluation of this parameter.

Since soft tissue edema and bone marrow edema were most frequently found in our dataset, fluid-sensitive sequences with fat suppression (like STIR) should be part of every MRI protocol. The regression of bone marrow edema on MRI is—apart from the clinical situation—the most important factor for the decision to cease off-loading treatment in these patients [19, 21]. Surprisingly, the intensity of bone marrow edema did not correlate with the duration of off-loading therapy. This may be explained by the bone marrow edema often persisting longer than the underlying inflammatory process and therefore may appear worse than the clinical situation [19]. All patients were diagnosed with an active Charcot foot at the time of MRI. That explains the high rate of soft tissue edema, which is pronounced during the early stage of Charcot foot and decreases typically within the first 4 weeks during off-loading treatment [19].

Subchondral cysts were frequently observed in our patients, present in region III in almost 50% of feet. Since these cysts are a typical finding of a long-standing Charcot foot [3, 24], we speculate that many patients in our study have had an active Charcot foot for at least some months prior to investigation. Some may have had a re-activation of inflammation in a chronic Charcot foot, which can occur in up to 23% of Charcot feet [18].

**Fig. 4:**
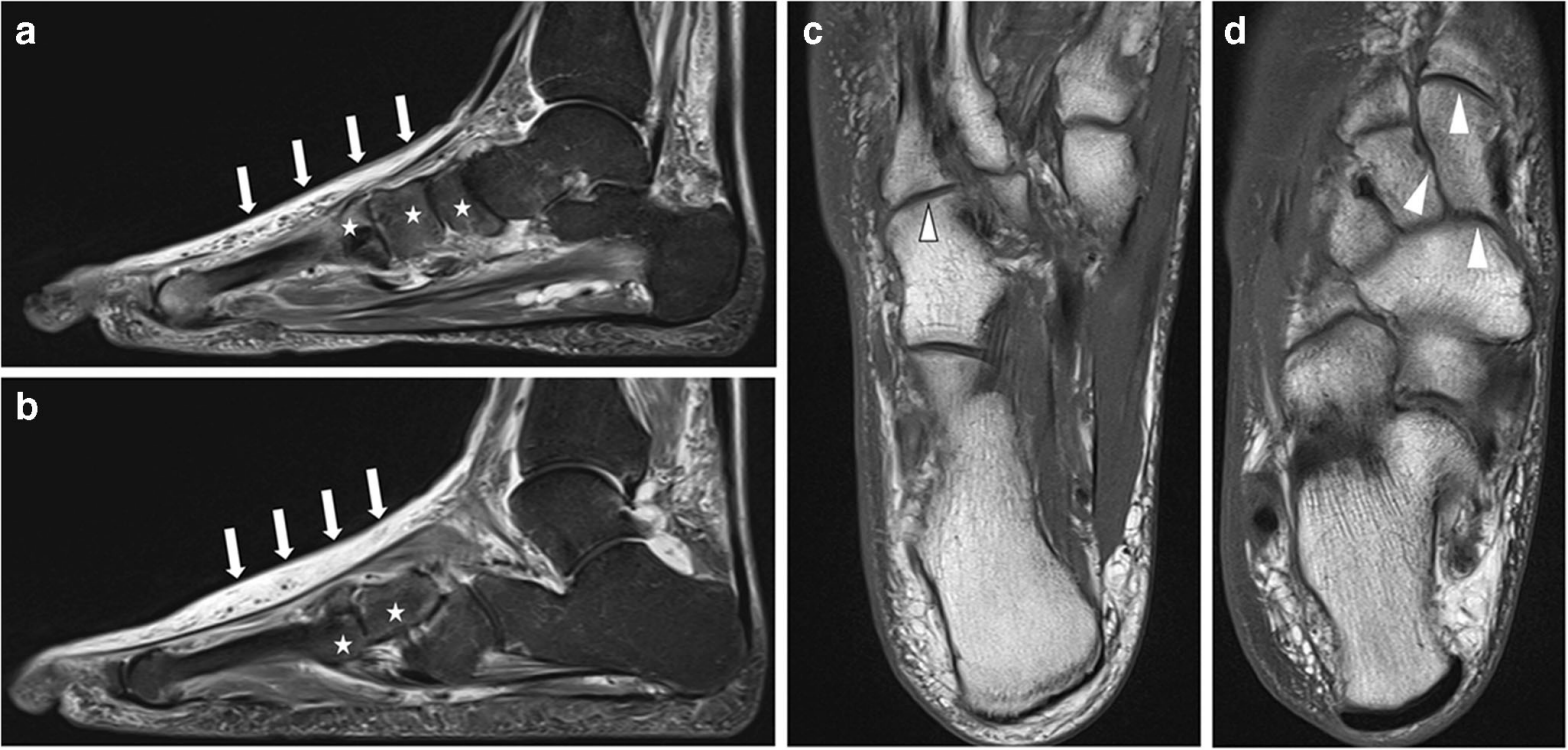
Eighty-four-year-old male patient (right foot) with Balgrist Score of 8 points and off-loading therapy of 64 days. **a**, **b** Sagittal STIR sequence showing severe soft tissue edema (white arrows) and some bone marrow edema (0–33%; asterisk) in region II and III. **c**, **d** No joint destruction is seen on transverse T1-weighted images (white arrowheads). No fracture was found

**Fig. 5:**
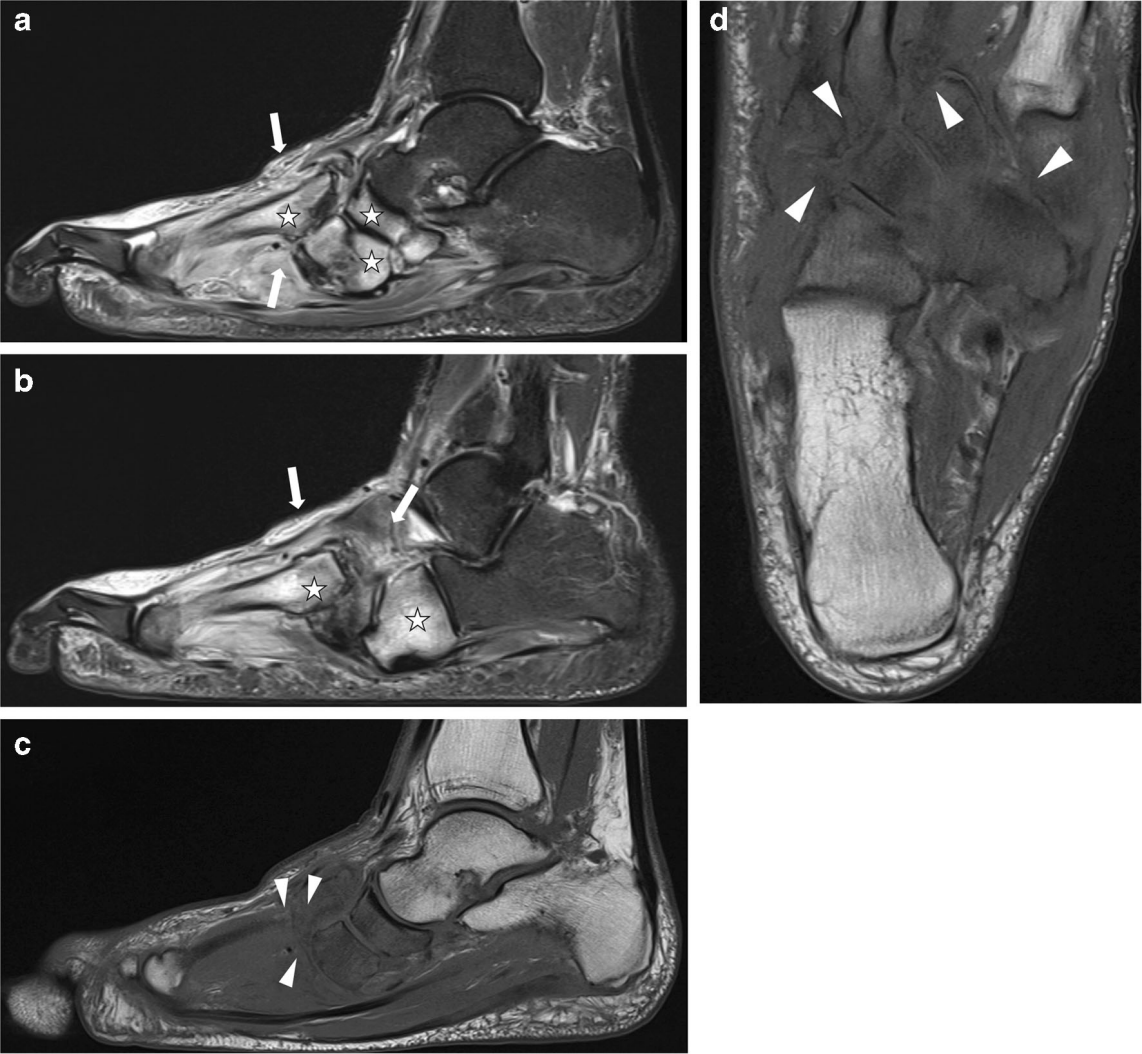
Forty-eight-year-old male patient (right foot) with Balgrist Score of 12 points and off-loading therapy of 375 days. **a**, **b** Sagittal STIR sequence. Soft tissue edema (white arrows) was rated as severe in region II and moderate in region III. Bone marrow edema (asterisk) was rated as severe (> 66%) in region II and moderate (34–66%, asterisk) in region III. Sagittal T1 sequence (**c**) and transverse T1 sequence (**d**) show moderate joint destruction (34–66%, white arrow heads) in region II and no destruction in region III. No fracture was seen

We searched for predictive parameters in all five foot regions and found an acceptable predictive value for the Whole Foot Score for a duration of ≥ 90 days for off-loading treatment. To create a score that is practical for daily use by radiologists and clinicians, we chose to simplify the Whole Foot Score by using only four parameters in the two most affected regions II and III: The Balgrist Score can be quickly calculated and has a sensitivity (66%) and specificity (72%) similar to those of the Whole Foot Score. Two typical examples of patients evaluated using the Balgrist Score are presented in Figs. 4 and 5. We are aware that the Balgrist Score has limited accuracy. However, since no other clinical or radiological parameters are currently available for the prediction of prolonged off-loading therapy in these patients, the score may help clinicians to prepare patients with a score ≥ 9 points right from the initiation of therapy that a prolonged cast treatment may be necessary.

An increasing trend in recent literature promotes an earlier surgical correction of the Charcot foot deformity with joint fusion (arthrodesis) to achieve an improved patient-perceived quality of life compared to the traditional off-loading therapy [15, 25]. The Balgrist Score may help the surgeon to choose the best fitting treatment strategy for each patient.

The interreader reliability was excellent in almost all MR regions and better than the ICC values assessed in the MR study by Meacock et al. [21] for bone marrow edema (ICC between 0.77 and 0.93) and fracture assessment (ICC between 0.49 and 0.70). Chantelau and Grützner proposed a new MRI classification [26] with two severity grades (presence or absence of bone marrow edema) and two stages (active stage and inactive stage) but did not report interreader reliability or clinical correlation. Our study showed that the Balgrist Score can be reliably assessed by radiologists as well as clinicians, as the interreader reliability between the orthopedic surgeon and the radiologists was excellent for almost all parameters and all regions. The only area with fair interreader reliability (comparing all three readers) was in region V, which is the least commonly affected and was therefore not included in the Balgrist Score.

This study has several limitations: due to the retrospective study design, we relied on the clinical documentation for patients with non-compliance for off-loading treatment, and the time point of the MR examination (in relation to the disease process) was not standardized. The predictive value of MRI findings might be higher when examined strictly within the first 2 weeks after symptom onset and is worthy of further study. However, in practical terms, this is difficult to achieve in our clinic since most patients are referred weeks or months after symptom onset with a missed diagnosis of Charcot foot. The calculation of the Balgrist Score is only possible in patients with Charcot-related changes in regions II and III, but this should not be a major limitation since few patients have Charcot-related changes in one region alone or in regions other than II and III. Other clinical parameters, such as blood parameters, might be important factors for the prediction of off-loading duration, but they were not part of this investigation. Recent studies found an additive value for diffusion-weighted imaging in the evaluation of the Charcot foot and differentiation of Charcot activity–related bone marrow edema and osteomyelitis [27, 28]. We did not evaluate diffusion-weighted sequences in this retrospective study since our institutional protocol does not currently include these sequences. However, the value of these additional advanced imaging parameters on therapy success should be evaluated in future studies.

In conclusion, the Balgrist Score is a new scoring system for daily routine MRI assessment of the Charcot foot with excellent interreader reliability. The Balgrist Score can help to identify patients with presumably prolonged off-loading treatment ≥ 90 days.

## Electronic supplementary material

ESM 1 (DOCX 1.52 mb)
