# Supplementary material for: The “Balgrist Score” for evaluation of Charcot foot: a predictive value for duration of off-loading treatment
Source: Skeletal Radiol. 2020 Jul 23;50(2):311–20. doi: 10.1007/s00256-020-03541-6 (PMC7736011; doi:10.1007/s00256-020-03541-6)
Supplement: Supplementary file 1 — (DOCX 1.52 mb) [file 256_2020_3541_MOESM1_ESM.docx]

**Supplemental Material**

Figure S1: Balgrist Standard Charcot MRI-Protocol


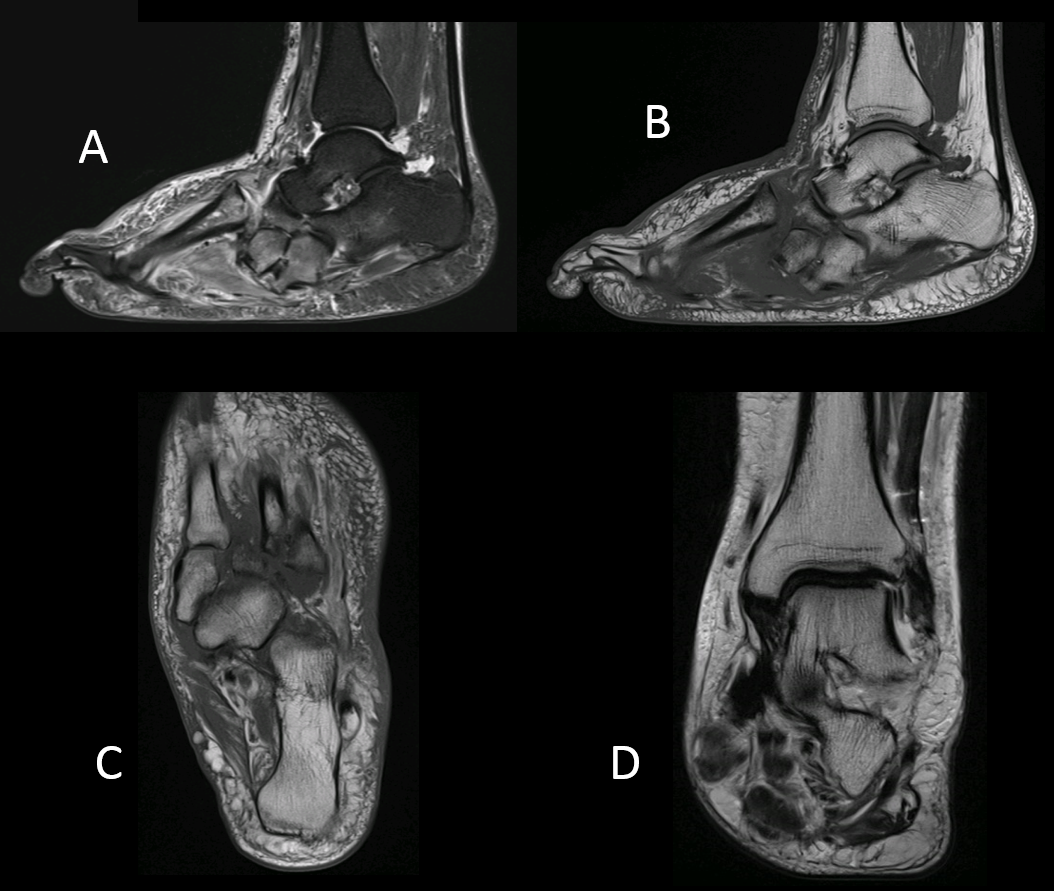


A: Sagittal STIR sequence (3 mm) whole foot; B: Sagittal T1 Sequence (3 mm)

whole foot; C: Transverse T1 Sequence hinfoot (3 mm) including tarsometatarsal

joints; D: Coronar T2 sequence (3 mm) hindfoot.

Figure S2: Soft tissue edema classification


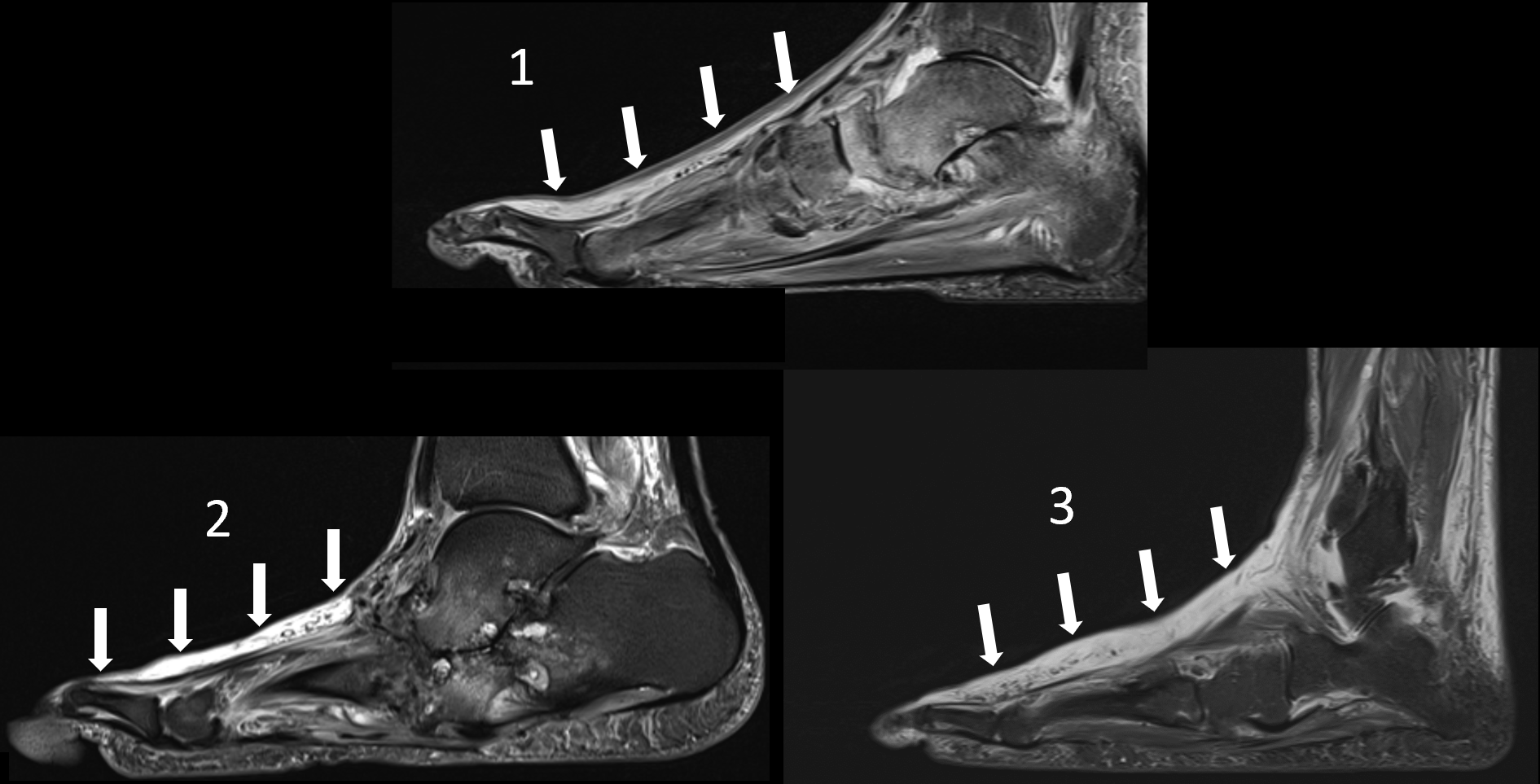


Showing degree (1 = mild, 2 = moderate and 3 = severe) of subcutaneous soft tissue edema (white arrows) in region II in three patients with Charcot foot.

Table S1

| **Overall Agreement per Item for Reader 1 & 2 using Fleiss` kappa** | | | | | | | |
| --- | --- | --- | --- | --- | --- | --- | --- |
| Item |  | Kappa | Asymptotic |  |  | Asymptotic 95% Confidence Interval | |
|  |  |  | Standard Error | Z | Sig. | Lower Bound | Upper Bound |
| Region_I_soft_tissue_edema | Overall Agreement | .724 | .078 | 9.287 | .000 | .719 | .729 |
| Region_I_bone_edema | Overall Agreement | .604 | .113 | 5.357 | .000 | .597 | .612 |
| Region_I_bone_erosion | Overall Agreement | .734 | .124 | 5.915 | .000 | .726 | .741 |
| Region_I_fracture | Overall Agreement | .734 | .124 | 5.915 | .000 | .726 | .741 |
| Region_I_subchondral_cysts | Overall Agreement | .792 | .124 | 6.385 | .000 | .784 | .800 |
| Region_I_joint_destruction | Overall Agreement | .651 | .124 | 5.245 | .000 | .643 | .658 |
| Region_I_regional_manifestation | Overall Agreement | .617 | .101 | 6.119 | .000 | .611 | .624 |
| Region_II_soft_tissue_edema | Overall Agreement | .843 | .083 | 10.163 | .000 | .838 | .848 |
| Region_II_bone_edema | Overall Agreement | .846 | .078 | 10.890 | .000 | .841 | .851 |
| Region_II_bone_erosion | Overall Agreement | .875 | .073 | 11.974 | .000 | .870 | .879 |
| Region_II_fracture | Overall Agreement | .901 | .124 | 7.262 | .000 | .893 | .908 |
| Region_II_subchondral_cysts | Overall Agreement | .822 | .114 | 7.222 | .000 | .815 | .830 |
| Region_II_joint_destruction | Overall Agreement | .892 | .075 | 11.924 | .000 | .887 | .897 |
| Region_II_regional_manifestation | Overall Agreement | .825 | .078 | 10.585 | .000 | .820 | .830 |
| Region_III_soft_tissue_edema | Overall Agreement | .825 | .090 | 9.173 | .000 | .819 | .830 |
| Region_III_bone_edema | Overall Agreement | .795 | .082 | 9.680 | .000 | .790 | .800 |
| Region_III_bone_erosion | Overall Agreement | .822 | .079 | 10.402 | .000 | .817 | .827 |
| Region_III_fracture | Overall Agreement | .792 | .124 | 6.385 | .000 | .784 | .800 |
| Region_III_subchondral_cysts | Overall Agreement | .885 | .112 | 7.907 | .000 | .878 | .892 |
| Region_III_joint_destruction | Overall Agreement | .864 | .079 | 10.916 | .000 | .859 | .869 |
| Region_III_regional_manifestation | Overall Agreement | .813 | .082 | 9.967 | .000 | .808 | .818 |
| Region_IV_soft_tissue_edema | Overall Agreement | .758 | .096 | 7.908 | .000 | .752 | .764 |
| Region_IV_bone_edema | Overall Agreement | .781 | .087 | 8.925 | .000 | .775 | .786 |
| Region_IV_bone_erosion | Overall Agreement | .688 | .094 | 7.303 | .000 | .682 | .694 |
| Region_IV_fracture | Overall Agreement | 1.000 | .124 | 8.062 | .000 | .992 | 1.008 |
| Region_IV_subchondral_cysts | Overall Agreement | .844 | .115 | 7.339 | .000 | .837 | .851 |
| Region_IV_joint_destruction | Overall Agreement | .892 | .096 | 9.313 | .000 | .886 | .898 |
| Region_IV_regional_manifestation | Overall Agreement | .752 | .088 | 8.532 | .000 | .747 | .758 |
| Region_V_soft_tissue_edema | Overall Agreement | .614 | .107 | 5.754 | .000 | .607 | .621 |
| Region_V_bone_edema | Overall Agreement | .602 | .124 | 4.857 | .000 | .595 | .610 |
| Region_V_fracture | Overall Agreement | ----- | ------- | ------- | ------ | ------- | ------ |
| Region_V_regional_manifestation | Overall Agreement | .467 | .124 | 3.767 | .000 | .459 | .475 |
